# Vancomycin dosing design method considering risk factors for nephrotoxicity

**DOI:** 10.1186/s40780-025-00416-7

**Published:** 2025-02-21

**Authors:** Yoshihiko Matsuki, Yutaro Kozima, Megumi Yanagi, Ken-ichi Sako, Tamaki Watanabe, Nobuhiro Yasuno, Shigekazu Watanabe

**Affiliations:** 1 Center for Promotion of Pharmaceutical Education & Research, Teiyo University, Tokyo, Japan; 2 Department of Pharmacy, Kashiwa Kousei General Hospital, Ageo Medical Group, Ageo, Japan; 3 Department of Clinical Pharmacy, Nihon Pharmaceutical University, Saitama, Japan; 4 Laboratory of Hospital Pharmacy, Teikyo University, Tokyo, Japan; 5 Department of Pharmacy, Teikyo University Hospital, 2-11-1, Kaga, Itabashi-ku, Tokyo, 173-8605 Japan

**Keywords:** Vancomycin, Nephrotoxicity, Therapeutic drug monitoring, Target concentration intervention

## Abstract

**Background:**

Vancomycin (VCM) induces nephrotoxicity in a dose-dependent manner, and patients with risk factors for nephrotoxicity have been reported to develop nephrotoxicity even within the effective concentration range. In the present study, we investigated measures to set an appropriate AUCss for each case by assessing the risk of developing nephrotoxicity using logistic regression curves, separating patients into a High-risk group with risk factors associated with nephrotoxicity when VCM is used and a Low-risk group without risk factors.

**Methods:**

A multivariate logistic regression analysis was used to identify risk factors for nephrotoxicity. The AUCss threshold was selected by a CART analysis and ROC curves, and a logistic regression analysis was used to examine the relationship between AUCss and the probability of developing nephrotoxicity.

**Results and discussion:**

The incidence of nephrotoxicity was 31.7% (33/104) in the High-risk group and 13.0% (14/108) in the Low-risk group, and was significantly higher in the former (*p* = 0.001). The AUCss threshold was set at 575 mg·h/L for the High-risk group and 650 mg·h/L for the Low-risk group. The probability of developing nephrotoxicity in the High-risk group (104 patients) was high: AUCss 400 mg·h/L (16.8%), 500 mg·h/L (23.3%), and 575 mg·h/L (29.3%). The target concentration range was newly set at 400 ≤ AUCss < 500, suggesting that the target AUCss needs to be considered for each patient based on the balance between therapeutic efficacy and the prevention of adverse effects. The probability of developing nephrotoxicity in the Low-risk group (108 patients) was AUCss 500 mg·h/L (4.7%), 575 mg·h/L (8.4%), and 650 mg·h/L (14.6%). Since the Low-risk group has a high safety profile, the target concentration range was newly set at 400 ≤ AUCss < 650, suggesting the safe administration of the drug up to AUCss 650 mg·h/L while aiming for AUCss 600 mg·h/L from the initial dose design.

**Conclusion:**

In the present study, the risk of nephrotoxicity for each AUCss was quantitatively analyzed using logistic regression curves for the High- and Low-risk groups. This allowed for the proposal of strategic individual target concentrations based on the balance between risk and benefit.

## Background

Although most antibacterial agents subjected to therapeutic drug monitoring (TDM) have target therapeutic ranges established in the TDM guidelines for antibacterial agents, flexible target ranges based on patient conditions are expected to improve therapeutic outcomes. Vancomycin (VCM), a glycopeptide antimicrobial agent, is used as a first-line treatment for methicillin-resistant *Staphylococcus aureus* (MRSA) infection in many diseases [1]. Typical side effects of VCM include nephrotoxicity, which may lead to treatment modifications or discontinuation, failure to treat infections, and the worsening of life expectancy. TDM of VCM typically involves measuring its blood concentration at a specific time point and using that value to predict efficacy and side effects, thereby guiding the dosing regimen. However, blood concentrations may significantly vary due to the patient’s condition and the progression of treatment. Therefore, it is essential to closely monitor the patient’s status daily and appropriately control the blood concentration of VCM. The nephrotoxicity of VCM is dependent on cumulative exposure rather than on transient high concentrations. Therefore, evaluating the impact of VCM on renal function using the average area under the concentration-time curve (AUCss) during the dosing period is considered to provide more accurate predictions. Nephrotoxicity due to VCM manifests when the AUCss consistently exceeds 600 mg·h/L [2–4]. Therefore, dosing regimens are designed to adjust the AUCss to 400–600 mg·h/L based on blood concentration measurements [2, 5, 6]. However, depending on concomitant medications [7, 8], underlying diseases [9–11], and patient backgrounds [9, 12], the onset of nephrotoxicity may occur at lower concentrations, requiring more precise dosing management in patients at a high risk of developing nephrotoxicity. Although previous studies reported nephrotoxicity risk factors for VCM, few proposed individualized dosing designs based on these risk factors [8]. VCM is an antimicrobial agent that is used in many healthcare settings and, thus, it is clinically critical to consider appropriate dosing strategies that minimize the risk of nephrotoxicity. This enables the AUCss to be set for patients with risk factors, balancing safety and therapeutic efficacy.

In the present study, we investigated measures to set an appropriate AUCss for each case by analyzing the relationship between AUCss and the probability of developing nephrotoxicity using a logistic regression analysis, separating patients with and without risk factors for nephrotoxicity when using VCM.

## Methods

### Subjects

This single-center, retrospective study was conducted at Kashiwa Kosei General Hospital between April 2021 and November 2022. Selection criteria included adult patients aged 18 years and older who were treated with VCM for infections and for whom the AUCss was calculated by measuring peak and trough blood concentrations at two points. Patients excluded were those on hemodialysis, those on continuous renal replacement therapy, those who received treatment for 2 days or less, and those who had only one peak or trough blood concentration point drawn. TDM was performed for all patients with measured VCM blood concentrations, and dosing was adjusted to achieve an AUC of 400–600 mg·h/L.

### Measurement items

Clinical data were obtained from electronic records. The following items were measured: age, sex, height, weight, body mass index (BMI), a high dose of VCM (4 g/day), loading dose ≥ 25 mg/kg, initial dose (mg/kg), maintenance dose (mg/kg/day), date of the first blood concentration measurement (day), number of peak blood concentration measurements, number of trough blood concentration measurements, peak and trough blood concentrations during VCM administration, number of days of administration, general blood tests [white blood cell count, platelet count], blood biochemical tests [serum albumin, aspartate aminotransferase increase (AST), alanine aminotransferase increase (ALT), blood urea nitrogen (BUN), serum creatinine (Cr), and C-reactive protein], and creatinine clearance (Ccr) calculated by the Cockcroft-Gault formula [13]. Concomitant medications that may impair renal function [tazobactam/piperacillin (TAZ/PIPC), aminoglycoside antibiotics (AGs), diuretics, non-steroidal anti-inflammatory drugs (NSAIDs), acetaminophen, angiotensin-converting enzyme inhibitors, angiotensin II receptor blockers, catecholamines (dopamine hydrochloride, dobutamine hydrochloride, adrenaline, and noradrenaline), immunosuppressive drugs, anticancer drugs, contrast agents, and amphotericin B] and comorbidities (heart failure, arrhythmia, renal diseases caused by underlying conditions, chronic hepatic disease, diabetes mellitus, malignant tumors, and sepsis) were confirmed. The criteria for selecting concomitant medications were the medications used during the VCM treatment period, while those for anticancer agents also included the period of drug withdrawal during the cancer chemotherapy course in the concomitant treatment period.

### Drug blood concentration assays and pharmacokinetic (PK) analysis

Blood samples were collected immediately before the next dose (trough value) and 2 h after the end of administration (peak value) at the steady state (3–5 days after the start of administration, 2–5 days after a dose change). Drug blood concentrations were analyzed in serum by a homogeneous enzyme immunoassay (measurement range: 2.0–50.0 µg/mL) using the Emit^®^2000 Vancomycin Assay from Siemens Healthcare Diagnostics Co. When measuring patient samples with VCM concentrations ≥ 50.0 µg/mL, samples were diluted 2 or 3 times using distilled water or the zero concentration of Emit2000 Vancomycin Calibrators, as described in the official manual of Siemens Healthcare Diagnostics, before measurements. Internal validation at our facility also confirmed the reproducibility and accuracy of measurements using the dilution method. Individual PK parameters for each patient were estimated by Bayesian methods using measured blood concentrations. Population PK parameters for Japanese patients were adapted from the study by Yasuhara et al., with the limitation of CLcr ≤ 85 mL/min being changed to 120 mL/min [14] (Table 1). CLcr was calculated based on Cr using the Cockcroft-Gault equation. The AUCss for each patient was calculated by dividing the dose of VCM administered during the dosing period (Dose) by estimated individual clearance (CL).

**Table 1:** Population pharmacokinetics parameters of VCM in Japanese adult patients used for bayesian estimation

Population pharmacokinetics	Interindividual variability
CL (mL/min) = 0.797 × CLcr	ω_CL_ = 38.5%
(CLcr ≤ 120 mL/min) ^†)^	
K_12_ (h^− 1^) = 0.525	ω_k12_ = 50.0%
K_21_ (h^− 1^) = 0.213	ω_k21_ = 28.6%
Vss (L) = 60.7	ω_Vss_ = 25.4%
	Intra-individual variability
	δ = 23.7%

CL: VCM creatinine clearance, k_12_ and k_21_: transter rate constants, Vss: steady-state volume of distribution

^†)^ The restriction of CLcr > 85 mL/min was changed to 120 mL/min and applied

## Diagnostic criteria for nephrotoxicity

In the present study, the diagnostic criteria for nephrotoxicity were defined by the Acute Kidney Injury Network Classification [15]. Patients with Cr ≥ 0.3 mg/dL or ≥ 50% increase from the pre-treatment level in at least two consecutive measurements during the VCM treatment were considered to be nephrotoxic [2]. During the observation period, the highest Cr value up to 3 days before the start of VCM administration was compared with the highest Cr value up to 7 days after the end of VCM administration [8].

## Data analysis methods

The Mann-Whitney U-test was used to compare clinical parameters between the no nephrotoxicity and nephrotoxicity groups, while the χ^2^ test and Fisher’s exact probability test were employed for categorical variables. Through comparisons of clinical parameters, items with *P* < 0.1 were extracted based on previous studies to identify factors closely related to VCM nephrotoxicity. Factors with Spearman’s rank correlation coefficient > 0.4 were evaluated for multicollinearity, and extracted items were considered accordingly. Extracted variables were optimized using the stepwise method. A multivariate analysis was then conducted, and factors with significant differences were identified as risk factors for nephrotoxicity. Patients were divided into a High-risk group with one or more risk factors for nephrotoxicity and a Low-risk group without risk factors.

The relationship between the probability of developing nephrotoxicity and AUCss was examined using a logistic regression analysis, classification and regression tree (CART) analysis, and receiver operating characteristic (ROC) curve analysis. CART and ROC curve analyses were used to examine AUCss thresholds that separate the High- and Low-risk groups into the no nephrotoxicity and nephrotoxicity groups.

The probability of developing nephrotoxicity was calculated using the intercept (β_0_) and regression coefficients (β_1_) from the logistic regression analysis. The specific formula is as follows.

$${\rm{P = 1 / (1 + }}{{\rm{e}}^{{\rm{ - (\beta_{0} + \beta_{1x1} + \beta_{2x2} + \rm{ \bullet \bullet \bullet + \beta_{nxn})}}}}}{\rm{)}}$$

Using this formula, the probability of developing nephrotoxicity was predicted based on the AUC.

To examine the incidence of nephrotoxicity in the High- and Low-risk groups, survival time curves were drawn using the Kaplan-Meier method, and differences in survival rates were compared using the Log-rank test. The Bonferroni method was applied for corrections. The Cox proportional hazard ratio (HR) survival model was used to evaluate HR for comparisons between groups. The significance of differences was set at 5%. The analysis software used were R 3.5.0 for Windows and JMP ver.9.03.

## Ethical considerations

The present study was approved by the Ethics Committee of Kashiwa Kosei General Hospital, was conducted in compliance with the Guidelines for the Appropriate Handling of Personal Information by Medical and Nursing Care Providers, and was based on data obtained through medical treatment at this hospital (Kashiwa Kosei General Hospital Approval number: 2300-23).

## Results

### Patient backgrounds and laboratory values at the start of treatment

**Fig. 1:**
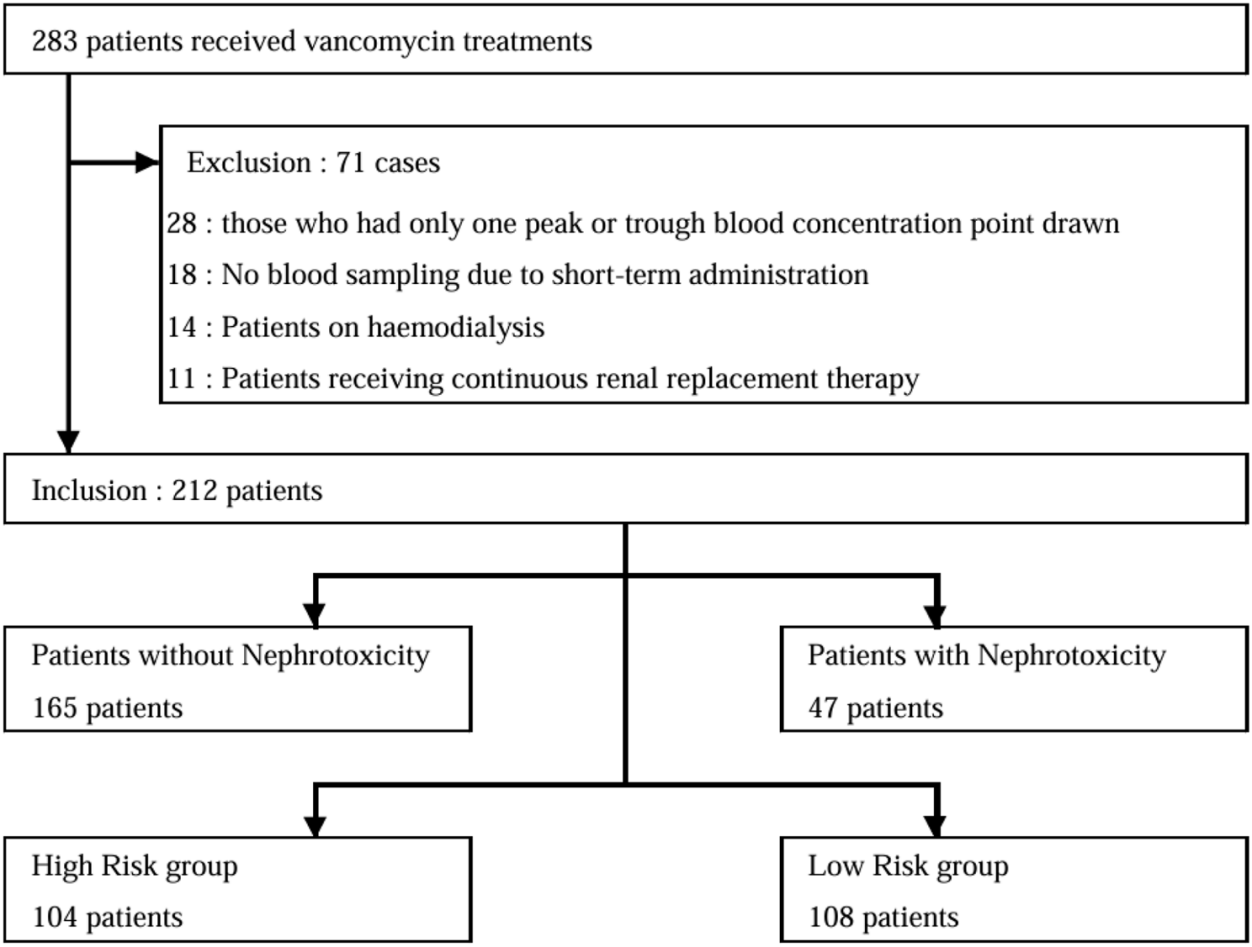
Process of enrollment of the study participants

The patient allocation process is shown in Fig. 1. A total of 212 eligible patients were divided into two groups: 165 (77.8%) in the no nephrotoxicity group and 47 (22.2%) in the nephrotoxicity group (Table 2). No significant differences were observed in age, height, weight, or BMI between the two groups. Blood biochemical tests showed that AST was significantly higher (*p* = 0.009) and ALT was also higher (*p* = 0.053) in the nephrotoxicity group. No significant differences were noted in BUN or Cr between the two groups. Regarding concomitant diseases and concomitant medications related to nephrotoxicity, significant differences were observed in chronic hepatic disease (*p* = 0.026), TAZ/PIPC (*p* = 0.038), AGs (*p* = 0.035), and diuretics (*p* = 0.008). The administration of NSAIDs was also more frequent in the nephrotoxicity group (*p* = 0.053).

**Table 2:** The clinical parameters of the no nephrotoxicity and nephrotoxicity groups

	Patients without Nephrotoxicity		Patients with Nephrotoxicity		*p*-value		
	(*n* = 165)		(*n* = 47)				
Age (years)	79	[34–96]	79	[19–98]	0.653^†)^		
Sex (M/F)	90/75		27/20		-		
Height (cm)	160	[137–180]	160	[137.5–178]	0.988^†)^		
Weight (kg)	51.3	[29.2-135.7]	49.1	[27.0-110.9]	0.633^†)^		
Ccr (mL/min)	49.6	[4.9–120]	54.4	[20.5–120]	0.385^†)^		
BMI	20.0	[12.5–49.8]	20.5	[11.7–45.0]	0.650^†)^		
Dehydration^‖)^	118	(71.5%)	33	(70.2%)	0.862^‡)^		
General blood tests							
WBC×10^3^ (/µL)	90.0	[1-405]	93.0	[3-242]	0.406^†)^		
Plt×10^4^ (/µL)	18.0	[0.2–66.6]	17.2	[1.5–59.7]	0.911^†)^		
Blood biochemical tests							
Alb (g/dL)	2.4	[1.3–4.3]	2.3	[1.3–3.7]	0.214^†)^		
T-Bil (mg/dL)	0.6	[0.2–9.5]	0.8	[0.2–5.6]	0.154^†)^		
AST (IU/L)	23.0	[6.0-324]	35.0	[10–155]	0.009^†)^		
ALT (IU/L)	17.5	[2.0-495]	26.0	[4.0-217]	0.053^†)^		
BUN (mg/dL)	20.1	[4.3–145]	20.8	[6.9–63.2]	0.948^†)^		
Cr (mg/dL)	0.74	[0.25–6.7]	0.63	[0.27–2.7]	0.428^†)^		
CRP (mg/dL)	7.5	[0.04–39.2]	7.8	[0.32–27.3]	0.817^†)^		
High dose of VCM (4 g/day)	2	(1.2%)	3	(6.4%)	0.074^§)^		
Cumulative Dose (g)	13.5	[2.75–82.5]	16.0	[3.0–65.0]	0.622^†)^		
Loading dose ≥ 25 mg/kg	103	(62.4%)	30	(63.8%)	0.860^‡)^		
Initial Dose (mg/kg)	26.0	[7.5–46.7]	25.6	[9.0-55.6]	0.180^†)^		
Maintenance Dose (mg/kg/day)	25.7	[3.5–63.9]	28.3	[5.4–62.3]	0.397^†)^		
Date of the first blood concentration measurement (day)	4	[3–10]	4	[3–8]	0.157^†)^		
Number of peak blood concentration measurements	1	[1–7]	2	[1–6]	0.130^†)^		
Number of trough blood concentration measurements	2	[1–7]	2	[1–6]	0.116^†)^		
Peak blood concentration (µg/mL)	24.7	[5.9–68.5]	29.0	[16.7-120.4]	< 0.0001^†)^		
Trough blood concentration (µg/mL)	15.4	[3.0-47.9]	19.3	[9.5–49.8]	< 0.0001^†)^		
Initial Blood Sampling Measured AUC (mg·h/L)	487	[237–1451]	628	[275–1975]	< 0.0001^†)^		
AUCss (mg·h/L)	551	[230–1036]	656	[287–1984]	< 0.0001^†)^		
Cumulative AUC (mg·h/L) ^¶)^	6001	[1011–37078]	5877	[1117–29657]	0.796^†)^		
Days of administration (days)	11.0	[3–43]	14.0	[3–47]	0.022^†)^		
Periods before the onset of nephrotoxicity (days)	11.0	[3–43]	11.0	[3–47]	0.437^†)^		
Dosing for more than 14 days	59	(35.8%)	24	(51.1%)	0.058^‡)^		
Comorbid disease							
Heart failure	53	(32.1%)	19	(40.4%)	0.289^‡)^		
Arrhythmia	32	(19.4%)	14	(29.8%)	0.127^‡)^		
Renal diseases caused by underlying conditions	30	(18.2%)	13	(27.7%)	0.154^‡)^		
Chronic hepatic disease	8	(4.8%)	7	(14.9%)	0.026^‡)^		
Diabetes mellitus	51	(30.9%)	12	(25.5%)	0.477^‡)^		
Malignant tumor	31	(18.8%)	6	(12.8%)	0.337^‡)^		
Sepsis	26	(15.8%)	6	(12.8%)	0.613^‡)^		
Concomitant medications^*)^							
TAZ/PIPC	14	(8.5%)	9	(19.1%)	0.038^‡)^		
Aminoglycosids^**#)**^	1	(0.6%)	3	(6.4%)	0.035^§)^		
Diuretic	59	(35.8%)	27	(57.4%)	0.008^‡)^		
NSAIDs	20	(12.1%)	11	(23.4%)	0.053^‡)^		
Acetaminophen	37	(22.4%)	12	(25.5%)	0.656^‡)^		
ACE inhibitors	2	(1.2%)	2	(4.3%)	0.214^§)^		
ARB	38	(23.0%)	8	(17.0%)	0.378^‡)^		
Catecholamine	28	(20.0%)	9	(19.1%)	0.728^‡)^		
Immunosuppressive drug	31	(18.8%)	9	(19.1%)	0.956^‡)^		
anticancer drug	7	(4.2%)	1	(2.1%)	0.871^§)^		
Contrast agent	1	(0.6%)	1	(2.1%)	0.395^§)^		

Median [range] ^†)^ Mann-Whitney’s *U* test ^‡)^ χ^2^ test ^§)^ Fisher’s exact test ^‖)^ Dehydration was defined as a blood urea nitrogen serum creatinine ratio > 20 ^¶)^ The cumulative AUC until the onset of nephrotoxicity during the vancomycin administration period ^**#)**^Gentamicin: 2Cases, Amikacin: 1 Cases, Streptomycin: 1 Cases ^*)^No patients were co-administered Amphotericin B

VCM: Vancomycin, ACE: Angiotensin converting enzyme inhibitors

## Blood concentrations, dosages, and days of administration of VCM

No significant differences were observed in the VCM cumulative dose (g), loading dose ≥ 25 mg/kg, initial dose (mg/kg), maintenance dose (mg/kg/day), date of the first blood concentration measurement (day), or number of blood concentration measurements between the no nephrotoxicity and nephrotoxicity groups (Table 2). The peak blood concentration was 24.7 µg/mL [5.9–68.5] (median [range]) in the no nephrotoxicity group and 29.0 µg/mL [16.7-120.4] in the nephrotoxicity group, with significantly higher values in the latter (*p* < 0.001).

Similarly, the trough blood concentration was 15.4 µg/mL [5.9–68.5] in the no nephrotoxicity group and 19.3 µg/mL [16.7-120.4] in the nephrotoxicity group, with significantly higher values in the latter (*p* < 0.001). The initial blood sampling measured AUC was 487 mg·h/L [237–1451] in the no nephrotoxicity group and 628 mg·h/L [275–1975] in the nephrotoxicity group, with significantly higher values in the latter (*p* < 0.001). Similarly, the AUCss was 551 mg·h/L [230–1036] in the no nephrotoxicity group and 656 mg·h/L [287–1984] in the nephrotoxicity group, with significantly higher values in the latter (*p* < 0.001). The cumulative AUC until the onset of nephrotoxicity during the VCM administration period was 6001 mg·h/L [1011–37078] in the no nephrotoxicity group and 5877 mg·h/L [1117–29657] in the nephrotoxicity group, with no significant difference between the two groups. The number of days of administration was 11.0 days [3–43] in the group without nephrotoxicity and 14.0 days [3–47] in the group with nephrotoxicity, which was significantly longer in the latter (*p*=0.022). Although no significant difference was observed in dosing for more than 14 days, it was more frequent in the nephrotoxicity group (*p* = 0.058). On the other hand, the period before the onset of nephrotoxicity did not significantly differ between the two groups (*p* = 0.437).

## Analysis of risk factors for nephrotoxicity

Comparisons of clinical parameters revealed that items with *P* < 0.1 were AST, ALT, a high dose of VCM (4 g/day), the peak value, trough value, initial blood sampling measured AUC, AUCss, days of administration (days), dosing for more than 14 days, TAZ/PIPC, AGs, diuretics, NSAIDs, and chronic hepatic disease. The peak value, trough value, and initial blood sampling measured values were excluded because they correlated with AUCss (correlation coefficients: peak value 0.403, trough value 0.498, initial blood sampling measured value 0.432). The number of days of administration was significantly higher in the nephrotoxicity group (*p* = 0.022). However, since there was no significant difference in the period before the onset of nephrotoxicity between the groups (*p* = 0.437), it was excluded. Based on previous studies, items closely related to VCM nephrotoxicity were identified as AST, ALT [16], a high dose of VCM (4 g/day) [17], AUCss [7], dosing for more than 14 days [18], TAZ/PIPC [7, 19], AGs [8], diuretics [7, 20], NSAIDs [21], and chronic hepatic disease [16]. Using the stepwise method, a multivariate logistic regression analysis of the selected factors identified AUCss, TAZ/PIPC, diuretics, and chronic hepatic disease as independent risk factors for nephrotoxicity (Table 3). Based on these results, patients were divided into two groups: a High-risk group with risk factors (104 patients) and a Low-risk group without risk factors (108 patients) (Table 4).

**Table 3:** Risk factors for nephrotoxicity

Risk factor	Univariate logistic regression analyses					Multivariate logistic regression analyses				
	Odds Ratio	95%CI			*p*-value	Odds Ratio	95%CI			*p*-value
AST (IU/L)	1.001	0.993	-	1.008	0.703	-	-	-	-	-
ALT (IU/L)	1.000	0.993	-	1.005	0.995	-	-	-	-	-
High dose of VCM (4 g/day)	5.556	0.895	-	43.17	0.065	6.971	0.519	-	93.57	0.143
AUCss (mg·h/L)	1.005	1.003	-	1.008	< 0.0001	1.006	1.004	-	1.009	< 0.0001
Dosing for more than 14 days	1.875	0.974	-	3.625	0.060	2.137	0.987	-	4.627	0.054
Chronic hepatic disease	3.434	1.143	-	10.13	0.029	4.273	1.285	-	14.21	0.018
TAZ/PIPC	2.555	0.998	-	6.282	0.050	4.627	1.562	-	13.70	0.006
AGs	11.18	1.394	-	229.0	0.023	10.12	0.752	-	136.4	0.081
Diuretic	2.425	1.259	-	4.742	0.008	2.189	1.012	-	4.736	0.047
NSAIDs	2.215	0.950	-	4.976	0.065	-	-	-	-	-

AUC: area under the concentration-time curve, VCM: Vancomycin, TAZ/PIPC: tazobactam/piperacillin, AGs: aminoglycoside antibiotics

The selection of factors was performed using the stepwise method

**Table 4:** The clinical parameters of Low Risk and High Risk groups

	Low Risk groups		High Risk groups		*p*-value		
	(*n* = 108)		(*n* = 104)				
Renal impairment	14	(13.5%)	33	(31.7%)	0.001^‡)^		
Age (years)	76	[33–95]	83	[19–98]	0.004^†)^		
Sex (M/F)	54/54		63/41		-		
Height (cm)	160	[138–180]	160	[137–175]	0.988^†)^		
Weight (kg)	51.7	[29.2-124.6]	48.9	[27.0-135.7]	0.451^†)^		
Ccr (mL/min)	55.3	[14.0-120]	46.9	[4.9–120]	0.045^†)^		
BMI	20.2	[12.5–47.5]	20.0	[11.7–49.8]	0.478^†)^		
Dehydration^‖)^	70	(64.8%)	81	(77.9%)	0.036^‡)^		
BUN (mg/dL)	18.7	[4.3–81.5]	22.3	[6.5–145.0]	0.004^†)^		
Cr (mg/dL)	0.72	[0.26–6.7]	0.74	[0.25–5.04]	0.505^†)^		
Initial Dose (mg/kg)	24.9	[9.0-46.7]	26.3	[7.5–55.6]	0.181^†)^		
Maintenance Dose (mg/kg/day)	28.8	[7.4–63.9]	23.7	[3.5–62.3]	0.004^†)^		
Peak blood concentration (µg/mL)	25.5	[12.1–56.8]	26.5	[8.5-120.4]	0.162^†)^		
Trough blood concentration (µg/mL)	15.6	[3.0-42.1]	16.4	[4.8–49.8]	0.060^†)^		
Initial Blood Sampling Measured AUC (mg·h/L)	492	[259–1291]	552	[237–1975]	0.095^†)^		
AUCss (mg·h/L)	572	[306–1217]	573	[230–1984]	0.500^†)^		
Cumulative AUC(mg·h/L) ^¶)^	5754	[1117–26181]	6402	[1011–37078]	0.349^†)^		
Days of administration (days)	11.5	[3–47]	13.0	[3–47]	0.196^†)^		
Periods before the onset of nephrotoxicity (days)	11	[3–41]	11.5	[3–47]	0.689^†)^		
Dosing for more than 14 days	36	(33.3%)	47	(45.2%)	0.077^‡)^		

Median [range] ^†)^ Mann-Whitney’s *U* test ^‡)^ χ^2^ test ^‖)^ Dehydration was defined as a blood urea nitrogen serum creatinine ratio > 20

^¶)^ The cumulative AUC until the onset of nephrotoxicity during the vancomycin administration period

## Comparisons between High- and Low-risk groups

The incidence of nephrotoxicity was 31.7% (33/104) in the High-risk group and 13.0% (14/108) in the Low-risk group, and was significantly higher in the former (*p* < 0.001) (Table 4). There were no significant differences in height, weight, or BMI between the High- and Low-risk groups. However, the High-risk group was significantly older (*p* = 0.004) and had lower Ccr (*p* = 0.045). There were no significant differences in Cr between the two groups. However, the High-risk group had significantly higher BUN levels (*p* = 0.004) and more patients with dehydration (*p* = 0.036). There were no significant differences in the initial dose of VCM (mg/kg), peak blood concentration, trough blood concentration, or initial blood sampling measured AUC between the two groups. The maintenance dose was 23.7 mg/kg/day [3.5–62.3] (median [range]) in the High-risk group and 28.8 mg/kg/day [7.4–63.9] in the Low-risk group, with the High-risk group receiving significantly lower doses (*p* = 0.004). However, the AUCss was 573 mg·h/L [230–1984] in the High-risk group and 572 mg·h/L [306–1217] in the Low-risk group, with no significant difference between the two groups. There were also no significant differences in the cumulative AUC until the onset of nephrotoxicity, the duration of administration, the period without nephrotoxicity, or administration beyond 14 days during the VCM treatment period between the two groups.

## Selection of AUCss thresholds based on the CART analysis and ROC curves

**Fig. 2:**
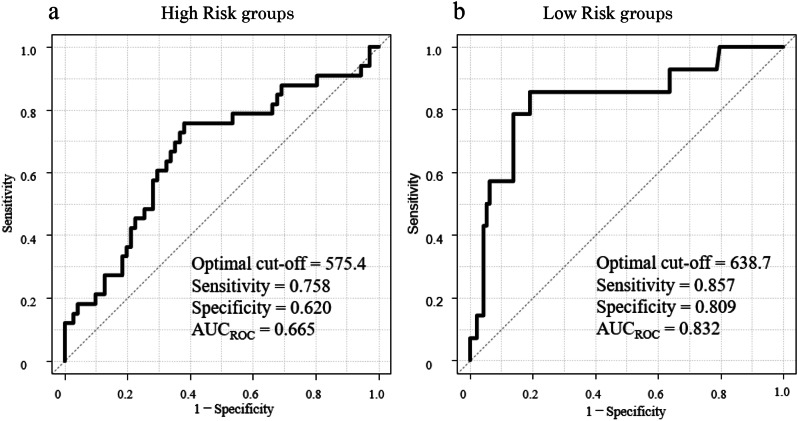
Receiver operating characteristic curve analysis of AUCss for predicting the clinical efficacy of VCM

AUCss thresholds were examined in the High- and Low-risk groups. In the CART analysis, the root node (node 0) was composed of two classes of nephrotoxicity: the no nephrotoxicity and nephrotoxicity groups, and a decision tree was constructed with AUCss as the variable. The results obtained showed that the threshold for dividing the root node in the High-risk group was 575.4 [AUCss ≥ 575.4 (27 patients in the no nephrotoxicity group and 25 patients in the nephrotoxicity group) and AUCss < 575.4 (44 patients in the no nephrotoxicity group and 8 patients in the nephrotoxicity group)]. The threshold for the Low-risk group was 638.7 [638.7 ≤ AUCss (18 patients in the no nephrotoxicity group and 12 patients in the nephrotoxicity group) and AUCss < 638.7 (76 patients in the no nephrotoxicity group and 2 patients in the nephrotoxicity group)]. ROC curves were used to select the threshold value that separates the no nephrotoxicity and nephrotoxicity groups. The threshold value was 575.4 (AUC0.67) for the High-risk group and 638.7 (AUC0.83) for the Low-risk group, which were consistent with the results of the CART analysis (Fig. 2). Based on these results, the AUCss threshold for the effective concentration range was defined as 575 for the High-risk group and 650 for the Low-risk group.

## Results of the logistic regression analysis

**Fig. 3:**
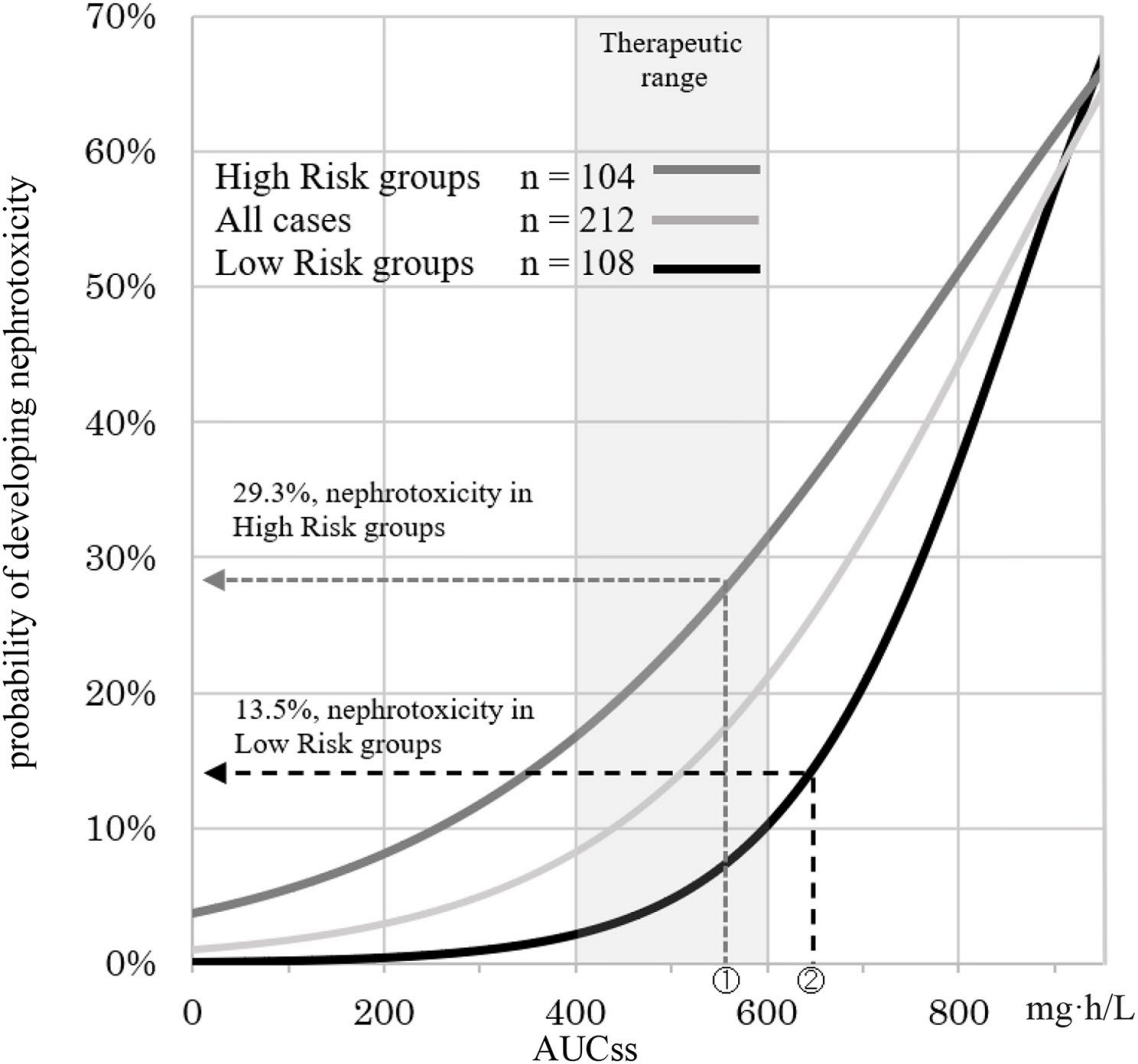
Graph of logistic regression analysis comparing High-risk and Low-risk groups. The currently recommended effective concentration range of vancomycin is shown in light gray. Threshold values based on ROC curves are indicated by dashed lines. ①The AUC threshold for the High-risk group was 575.4 mg·h/L. The probability of developing nephrotoxicity was 29.3%. ②The AUC threshold for Low-risk group was 638.7 mg·h/L. The probability of developing nephrotoxicity was 13.5%

A graph of the results of the logistic regression analysis comparing the High-risk group, Low-risk group, and all cases is shown in Fig. 3. Parameter estimates (standard errors) of the regression model for the High-risk group were − 3.248 (1.028) for the intercept and 0.004 (0.002) for the partial regression coefficient, with both being significant at *p* < 0.001. The parameter estimates (standard errors) of the regression model for the Low-risk group were − 7.11 (1.587) for the intercept and 0.008 (0.002) for the partial regression coefficient, with both being significant at *p* < 0.001. The parameter estimates (standard errors) of the regression model for all cases were − 4.60 (0.834) for the intercept and 0.005 (0.001) for the partial regression coefficient, with both being significant at *p* < 0.001.

AUCss (400, 500, 600) and AUCss thresholds (575, 650) in the effective concentration range were fit to the regression curves obtained by the logistic regression analysis for the Low-risk group, High-risk group, and all cases (Fig. 3; Table 5). The probabilities of developing nephrotoxicity within the effective concentration ranges were 16.8, 23.3, and 31.4% in the High-risk group; 2.1, 4.7, and 10.2% in the Low-risk group; and 8.2, 13.4, and 21.1% in all cases, respectively. The probabilities of developing nephrotoxicity at the AUCss threshold were 29.3 and 36.0% in the High-risk group, 8.4 and 14.6% in the Low-risk group, and 18.9 and 26.0% in all cases, respectively.

**Table 5:** Percentage of nephrotoxicity based on AUCss (*n* = 212)

AUCss (mg·h/mL)	Low Risk groups (*n* = 108)	High Risk groups (*n* = 104)	All cases (*n* = 212)
400	2.1%	16.8%	8.2%
500	4.7%	23.3%	13.4%
575	8.4%	29.3%	18.9%
600	10.2%	31.4%	21.1%
650	14.6%	36.0%	26.0%

AUC: area under the concentration-time curve

## Analysis of the incidence of nephrotoxicity in High- and Low-risk groups

**Fig. 4:**
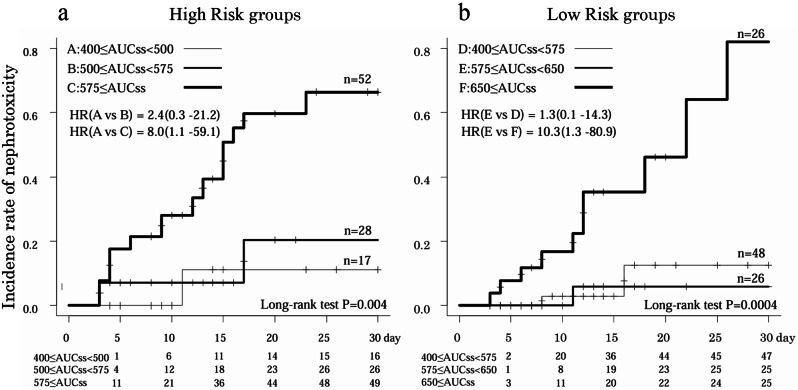
Kaplan-Meier survival plot comparing the incidence of nephrotoxicity in the High Risk and Low Risk groups. HR: Hazard Ratio

The High-risk group (400 ≤ AUCss < 500, 500 ≤ AUCss < 575, 575 ≤ AUCss) and Low-risk group (400 ≤ AUCss < 575, 575 ≤ AUCss < 650, 650 ≤ AUCss) were divided into three subgroups (Fig. 4).

In the High-risk group, there was no significant difference in the incidence of nephrotoxicity between 400 ≤ AUCss < 500 and 500 ≤ AUCss < 575 [HR 2.364; 95% CI 0.26–21.22; *p* = 0.44]. The incidence of nephrotoxicity was significantly higher for 575 ≤ AUCss than for 400 ≤ AUCss < 500 [HR 7.977; 95% CI 1.08–59.06; *p* = 0.042] and 500 ≤ AUCss < 575 [HR 3.374; 95% CI 1.17–9.74; *p* = 0.025] ( Fig. 4-a). In the Low-risk group, no significant difference was observed in the incidence of nephrotoxicity between 400 ≤ AUCss < 575 and 575 ≤ AUCss < 650 [HR 0.774; 95% CI 0.07–8.55; *p* = 0.835]. The incidence of nephrotoxicity was significantly higher for 650 ≤ AUCss than for 400 ≤ AUCss < 575 [HR 7.988; 95% CI 1.74–36.65; *p* = 0.008] and 575 ≤ AUCss < 650 [HR 10.320; 95% CI 1.32–80.86; *p* = 0.026] (Fig. 4-b).

## Discussion

In the present study, the risk of developing nephrotoxicity was assessed using logistic regression curves in the High-risk group with risk factors for nephrotoxicity when using VCM and the Low-risk group without risk factors, and measures to set the appropriate AUCss for each case were investigated. Based on comparisons of clinical parameters, the peak value, trough value, and initial blood sampling measured AUC were excluded as risk factors, and the AUCss was adopted. These parameters all depend on PK factors, such as the drug dosage, dosing interval, absorption, distribution, metabolism, and excretion, leading to correlations and multicollinearity. Correlation coefficients were 0.403 for the peak value, 0.498 for the trough value, and 0.432 for initial blood sampling measured AUC, all indicating correlations. Therefore, to avoid using these parameters simultaneously as risk factors, the AUCss was selected as the representative indicator. The relationship between the duration of VCM administration and nephrotoxicity was investigated. A comparison between the nephrotoxicity group and no nephrotoxicity group showed that the duration of administration was significantly longer in the former, while there was no significant difference in the period before the onset of nephrotoxicity between the groups. These results suggest that the administration of VCM continued even after the onset of nephrotoxicity in the nephrotoxicity group. Additionally, the lack of a significant difference in the no nephrotoxicity period suggests that the duration of VCM administration did not directly affect nephrotoxicity itself. On the other hand, the difficulties associated with controlling VCM blood concentrations with long-term use may contribute to the occurrence of nephrotoxicity. The AUCss was 573 mg·h/L [230–1984] (median [range]) in the High-risk group and 572 mg·h/L [306–1217] in the Low-risk group. Despite both groups being controlled within the effective concentration range, the High-risk group had a higher incidence of nephrotoxicity than the Low-risk group. Furthermore, the mean AUCss in the no nephrotoxicity group was 551 mg·h/L [230–1036], whereas it was significantly higher in the nephrotoxicity group at 656 mg·h/L [287–1984]. This result indicates that fluctuations in the AUCss associated with long-term administration increase the risk of nephrotoxicity. Therefore, considering fluctuations in and the difficulties associated with controlling blood concentrations with long-term administration, a duration of more than 14 days was selected as a potential risk factor. Based on these results, we identified the following factors as being closely related to VCM-induced nephrotoxicity: AST, ALT, a high dose of VCM (4 g/day), AUCss, dosing for more than 14 days, TAZ/PIPC, AGs, diuretics, NSAIDs, and chronic hepatic disease. After optimizing the model and performing a multivariate analysis, AUCss [7], TAZ/PIPC [19], diuretics [20], and chronic liver disease [16] were identified as independent risk factors for VCM-induced nephrotoxicity. These results are consistent with previous findings.

Diuretics [20] and hepatic disease [16] contribute to ischemia and cause nephrotoxicity by decreasing renal blood flow. The administration of VCM in the setting of a reduced circulating blood volume and renal blood flow due to infection may trigger acute kidney injury. TAZ/PIPC has been reported to increase the risk of VCM nephrotoxicity [19]. PIPC has the side effect of acute interstitial nephritis; however, the underlying mechanisms remain unclear. The combination of TAZ/PIPC and VCM is considered to cause tubular necrosis. The nephrotoxicity of AGs has been attributed to glomerular-filtered AGs being taken up by proximal tubular epithelial cells through endocytosis from the tubular lumen, resulting in tubular necrosis. Rybak et al. demonstrated that AGs caused nephrotoxicity at lower AUCss when administered with VCM than when given alone [8]. In the present study, 3 of 4 patients (Gentamicin: 2, Amikacin: 1) who received concomitant AGs developed nephrotoxicity; however, it was not identified as a risk factor. This result was due to the strong nephrotoxicity of AGs, resulting in a low number of cases combined with VCM, which, in turn, led to a low detection power.

Patients were divided into a High-risk group, with one or more independent risk factors for nephrotoxicity, and a Low-risk group, without any risk factors. The incidence of nephrotoxicity was compared between the two groups. The AUCss was 573 mg·h/L [230–1984] (median [range]) in the High-risk group and 572 mg·h/L [306–1217] in the Low-risk group, with blood concentrations controlled within the therapeutic range in both groups. However, the incidence of nephrotoxicity was significantly higher in the High-risk group (31.7%) than in the Low-risk group (13.0%). The incidence of nephrotoxicity in effective concentration range AUCss (400, 500, 600) were 2.1, 4.7, and 10.2% in the Low-risk group and 16.8, 23.3, and 31.4% in the High-risk group, respectively, which were higher in the latter. This result suggests the need for separate target concentration ranges for the two groups. Therefore, new target concentration ranges were defined for the High- and Low-risk groups using the threshold values obtained from the CART analysis and ROC curves as well as the probability of developing nephrotoxicity from logistic regression curves. In the High-risk group, the AUCss threshold was set at 575, and the probability of developing nephrotoxicity was compared in three groups: 400 ≤ AUCss < 500, 500 ≤ AUCss < 575, and 575 ≤ AUCss. The probability of developing nephrotoxicity in the 500 ≤ AUCss < 575 group did not significantly differ from that in the 400 ≤ AUCss < 500 group. However, the probability of developing nephrotoxicity in the logistic regression analysis was high at 23.3% at AUCss 500 and 29.3% at AUCss 575. Although the AUCss threshold for the High-risk group was 575.4, the discriminatory ability of the ROC curve was low at 0.665. The sigmoid curve obtained from the logistic regression analysis showed a smooth increase without abrupt changes at specific points, indicating a high rate of nephrotoxicity in the whole area. Therefore, the target concentration range appears to be 400 ≤ AUCss < 500 for safety reasons. A previous study reported that a VCM AUC24/MIC ratio of ≥ 505 was required for the treatment of severe infections [22]. The newly established target concentration range (400 ≤ AUCss < 500) is lower than the previously recommended range, necessitating caution to ensure its efficacy. Therefore, if the treatment effect is inadequate, a dose increase up to AUCss 505 is recommended and renal function needs to be carefully monitored with frequent measurements of blood levels. Additionally, if the treatment effect remains inadequate, switching to other anti-MRSA agents is suggested.

On the other hand, the AUCss threshold was set to 650 in the Low-risk group, and the probability of developing nephrotoxicity was compared among three groups: 400 ≤ AUCss < 575, 575 ≤ AUCss < 650, and 650 ≤ AUCss. The results obtained showed no significant difference in the probability of developing nephrotoxicity between 575 ≤ AUCss < 650 and 400 ≤ AUCss < 575.

The probability of developing nephrotoxicity by the logistic regression analysis was 8.4% for AUCss575 and 14.6% for AUCss650. In previous studies, the probability of developing nephrotoxicity due to VCM ranged between 12 and 48% [23, 24]. The AUCss threshold for the Low-risk group was 638.7, and the AUC of the ROC curve was 0.832, indicating a high discriminatory ability. Additionally, the sigmoid curve obtained from the logistic regression analysis showed an initial gradual increase, with a sharp elevation in the probability of developing nephrotoxicity near the threshold. Since the Low-risk group had a high safety profile, the target concentration range was newly set at 400 ≤ AUCss < 650, suggesting the safe administration of the drug up to AUCss650 while aiming for AUCss600 from the initial dose design. The upper limit of the target concentration range was considered to be 650 from the viewpoint of safety because the probability of developing nephrotoxicity was significantly higher at 650 ≤ AUC.

The increased probability of developing nephrotoxicity due to VCM was previously shown to be confounded by the concomitant use of nephrotoxic drugs, hemodynamic changes, and the effects of underlying diseases [25]. Although VCM is not classified as a nephrotoxic drug in the Kidney Disease Improving Global Outcomes acute kidney injury guidelines, the present study indicates that VCM itself is associated with a risk of nephrotoxicity [26].

The present results may contribute to the individualization of VCM dosing plans for the treatment of infectious diseases. As mentioned in the Introduction, the recommended effective concentration range for VCM is AUCss 400–600 mg·h/L for efficacy and safety [2]. However, the present study demonstrated that the probability of developing nephrotoxicity was high in the nephrotoxicity group, even within the effective concentration range, and, thus, a target AUCss needs to be set for each patient in consideration of the balance between treatment efficacy and the prevention of side effects. Holford et al. recommended a Target Concentration Intervention (TCI) as an alternative strategy to TDM [27]. TCI focuses on achieving specific target drug concentrations tailored to each patient’s condition. This approach involves establishing target drug concentrations that maximize therapeutic effects while minimizing side effects, and then adjusting the dosage to reach these concentrations. TCI utilizes PK models that take into account individual factors, such as the patient’s weight, age, renal function, and hepatic function. This enables the provision of optimal therapeutic effects for each patient while minimizing the risk of adverse effects. On the other hand, the concept of a therapeutic range for TDM has been reported to reduce the expected clinical benefit to patients because measured values below the lower end of the range, within the range, and above the upper end are classified as ‘sub-therapeutic’, ‘therapeutic’, and ‘toxic’, respectively, leading to uniform dosing recommendations based on measured blood levels [27, 28]. In the present study, the target concentration range was divided into High- and Low-risk groups, and a logistic regression curve was used to quantify the risk of nephrotoxicity for each AUCss. This allowed for proposals of strategic individual target concentrations based on the balance between risk and benefit. This approach aligns with the principles of TCI, which utilizes PK models that consider individual patient factors.

The novelty of this study lies in the identification of risk factors for nephrotoxicity in the design of VCM dosing and the establishment of optimal AUCss for each risk group. VCM is an antimicrobial agent used in many healthcare facilities and, thus, setting appropriate guidelines to reduce the risk of nephrotoxicity is extremely important. Previous studies reported the risk of nephrotoxicity with the administration of VCM, and the present study proposes an individualized dosing strategy based on specific risk factors. Furthermore, by examining the incidence of nephrotoxicity in detail in patients divided into High- and Low-risk groups, we provide useful insights for personalized medicine. This study is unique in that it classifies VCM into High- and Low-risk groups based on the presence or absence of risk factors and proposes a target concentration range appropriate for each group.

The present study has several limitations that need to be addressed. The number of cases in this study was small at only 212. In addition, the number of patients who received the combination of AGs and VCM, which is highly nephrotoxic, was small (only 4). Future multicenter studies on a more diverse patient population are needed. Since this study focused on reducing the risk of nephrotoxicity, the evaluation of the efficacy of VCM was insufficient. Therefore, therapeutic effects within the proposed target concentration range need to be confirmed. The 7-day monitoring of renal function after the cessation of VCM administration was useful for evaluating short-term changes in renal function, but was considered to be insufficient for assessing long-term effects. In the future, follow-up periods of 3 months or longer will be necessary to evaluate the risk of acute kidney injury progressing to chronic kidney disease. Although the group with nephrotoxicity risk factors was classified as the High-risk group, the probability of developing nephrotoxicity may differ between patients with a single risk factor and those with multiple risk factors. The regression curve obtained from the logistic regression analysis suggested a gradual increase in the incidence of nephrotoxicity from the Low-risk line to the High-risk line, according to the level of risk. Conducting large-scale multicenter collaborative studies and securing a sample size that maximizes detection power will allow for a detailed examination of the incidence of nephrotoxicity according to each risk factor and combinations of multiple risk factors. However, the development of nephrotoxicity largely depends on the patient’s condition. Therefore, it is crucial to understand the condition of patients with risk factors and the probability of developing nephrotoxicity at each AUCss, while frequently measuring blood concentrations and carefully monitoring renal function.

In recent years, higher doses have been required to prevent hyposensitization to VCM [29]. The development of prerenal acute renal failure due to septic shock or endotoxin shock is common in the ICU. Difficulties are associated with establishing whether renal failure in severe cases is due to prerenal renal failure caused by renal ischemia or drug-induced acute kidney injury. Therefore, a prospective study with data that refutes renal ischemia is needed to clarify the risk factors for nephrotoxicity. The validity of this method will be confirmed in the future by increasing the number of patients and tracking treatment outcomes in the Low- and High-risk groups.

## Conclusions

In the present study, the target concentration range was divided into High- and Low-risk groups, and the risk of nephrotoxicity for each AUCss was quantitatively analyzed for each group using a logistic regression curve. The AUCss threshold for the High-risk group was 575 mg·h/L. Due to the high probability of developing nephrotoxicity (16.8% at AUCss 400 mg·h/L, 23.3% at AUCss 500 mg·h/L, and 29.3% at AUCss 575 mg·h/L), the target concentration range was set at 400 ≤ AUCss < 500. In the high-risk group, the target AUCss needs to be set in consideration of the balance between the efficacy of treatment and the prevention of side effects in each patient. The AUCss threshold for the low-risk group was set at 650 mg·h/L. Due to the high safety profile, with probabilities of developing nephrotoxicity of 4.7% at AUCss 500 mg·h/L, 8.4% at AUCss 575 mg·h/L, and 14.6% at AUCss 650 mg·h/L, the target concentration range was set at 400 ≤ AUCss < 650. In the low-risk group, it is suggested that the initial dosing design should aim for an AUCss of 600 mg·h/L, while safely allowing administration up to an AUCss of 650 mg·h/L. This method enables not only the individualization of dosage and administration, but also the setting of target concentrations that take into account the risk factors of each patient. This method will lead to safer and more effective drug therapy.

## Data Availability

Data supporting the results of this study are available from the corresponding author upon reasonable request.

## Abbreviations

VCM: Vancomycin
TDM: Therapeutic drug monitoring
MRSA: Methicillin-resistant *Staphylococcus aureus*
AUC: Area under the concentration-time curve
BMI: Body mass index
AST: Aspartate aminotransferase
BUN: Blood urea nitrogen
Cr: Serum creatinine
TAZ/PIPC: Tazobactam/piperacillin
AGs: Aminoglycoside antibiotics
PK: Pharmacokinetic
CL: Clearance
CART: Classification and regression tree
ROC: Receiver operating characteristic
MIC: Minimum inhibitory concentration
